# Physicochemical Changes of Air-Dried and Salt-Processed *Ulva rigida* over Storage Time

**DOI:** 10.3390/molecules24162955

**Published:** 2019-08-15

**Authors:** Valentina F. Pinheiro, Catarina Marçal, Helena Abreu, José A. Lopes da Silva, Artur M. S. Silva, Susana M. Cardoso

**Affiliations:** 1 QOPNA & LAQV-REQUIMTE, Department of Chemistry, University of Aveiro, 3810-193 Aveiro, Portugal; 2 ALGAplus, Produção e Comercialização de Algas e seus Derivados, Lda., 3830-196 Ílhavo, Portugal

**Keywords:** *Ulva* sp., sea lettuce, color, texture, minerals, fatty acids, nutritional, brining, dry-salting, air-drying

## Abstract

The impact of air-drying at 25 °C, brining at 25%, and dry-salting (at 28% and 40%) on the quality and nutritional parameters of *Ulva rigida* were evaluated over six months of storage. Overall, the main changes occurred in physical aspects during storage time, with *U. rigida* intensifying its yellow/browning tones, which were more evident in salt-treated samples. The force necessary to fracture the seaweed also increased under all the preservative conditions in the first month. Conversely, the nutritional parameters of *U. rigida* remained stable during the 180 days of storage. All processed samples showed a high content of insoluble and soluble fibers, overall accounting for 55%–57% dw, and of proteins (17.5%–19.2% dw), together with significant amounts of Fe (86–92 mg/kg dw). The total fatty acids pool only accounted for 3.9%–4.3% dw, but it was rich in unsaturated fatty acids (44%–49% total fatty acids), namely palmitoleic (C16:1), oleic (C18:1), linoleic (C18:2), linolenic (C18:3), and stearidonic (18:4) acids, with an overall omega 6/omega 3 ratio below 0.6, a fact that highlights their potential health-promoting properties.

## 1. Introduction

Seaweeds, i.e., marine macroalgae (including Chlorophyta, Rhodophyta, and Ochrophyta/Phaeophyceae), are considered one of the non-animal foods of the future due to their ability to grow without using arable land or freshwater resources, combined with their recognized richness in valuable nutrients and phytochemicals, including proteins with high nutritional value, bioactive peptides, insoluble and soluble fibers, polyunsaturated fatty acids, minerals, vitamins, and polyphenols [1,2,3,4,5].

The direct consumption of macroalgae as food is still incipient in Western countries when compared to the Asiatic countries, but this trend is changing over the past years, mostly based on health claims associated with their regular consumption [6]. Indeed, food and nutraceutical industries have grown interest in introducing macroalgae as an ingredient in functional foods, and the number of products containing this “new ingredient” launched on the market is growing fast, particularly in Europe [7]. The global functional food market, evaluated at about $168 billion in 2013 and estimated to reach $305.4 billion by 2020 [8], is one of the market opportunities for the direct application of seaweeds, of purified extracts, or purified fractions.

However, the use of characteristic European macroalgae as a food ingredient faces huge challenges, that go from the sustainable production of biomass to hold the market development without disrupting marine resources and many others, directly or indirectly linked to it. Among direct implications to high biomass production, fast and controlled preservation methods will be required. In fact, seaweeds are naturally highly perishable due to their high water content (60% to 94%) [1], requiring the application of preservative methodologies to expand their shelf-life and to retain sensory, nutritional, and bioactive attributes. 

Among preservative methods, thermal treatments in forced air tunnels are currently the most applied in the industry to macroalgae, being recognized for generating dehydrated products with extended shelf-life and guaranteeing a high processing capacity. The main drawback of this method, besides energy costs, is associated with possible changes in the food matrix, which often impact product quality and sensory characteristics and might affect consumers approval. In addition, macroalgae are also often processed by non-thermal treatments, including freezing and salting, which are able to retard microorganism growth and lipid oxidation events by lowering the temperature or promoting osmotic dehydration through the use of common salt (sodium chloride), respectively [2,9,10].

*Ulva* sp. is widely distributed throughout the world and one of the two Chlorophyta genera allowed for consumption as vegetables and condiments in Europe [11], but still, the scientific knowledge about the impact of preservative processing in this genus is quite limited and only performed at a laboratory scale. In particular, Robic et al. [12] evaluated the impact of oven-drying (50 and 70 °C) on the yield of recovery and physicochemical features of ulvans, obtained from wild *Ulva rotundata*, while Rodrigues et al. [13] reported the influence of oven-drying (30 and 40 °C) on the rehydration ratio and water holding ability of *Ulva lactuca*. In turn, Uribe et al. [14] evaluated the effect of distinct drying methods, namely freeze-, vacuum-, solar-, and convective drying (at 70 °C), on the surface color of wild *Ulva* spp. from Chile, together with changes in the levels of phytochemicals and the profile of amino acids and fatty acids. Moreover, our group has recently evidenced the effect of distinct oven-drying temperatures (25, 40 and 60 °C) on quality parameters and recovery yield of valuable compounds (pigments, phenolic compounds, and polysaccharides) from *U. rigida*, as compared to freeze-drying [15]. 

Thus, the aim of the present work was to understand the impact of distinct preservative methods applied at the industrial level, namely air-drying in a convective dryer, dry-salting, and brining on the features of *U. rigida*, also considering storage time over six months.

## 2. Results

### 2.1. Moisture Content

The moisture content of unprocessed *U. rigida* was 80% (data not shown), which upon air-drying in a convective dryer at 25 °C for 16 h reached 14%. As expected, salt-processing also caused a significant decrement of *Ulva’s* water content, which was set to about 71% on brining and dry-salting (at 28%) and to 60% on dry-salting (at 40%), as shown in Table 1. 

The gathered data also allow us to conclude that the mean moisture value of dehydrated *U. rigida* did not vary significantly over 180 days, indicating that the storage conditions were adequate to maintain this parameter stable in the air-dried samples. A similar trend was also observed for the brined and dried-salted samples at 40%, suggesting that under these conditions, the osmotic equilibrium was reached in the first 4 h (time interval between collection and analysis of the sample after arrival at the laboratory), though they were maintained at low temperatures. In turn, osmotic balance in dry-salting at 28% samples was achieved later on, since values at t0 were higher than those measured at t30 (70.9% and 61.9%, respectively).

### 2.2. Superficial Color

Surface color is a quality attribute of food that is commonly affected by processing and storage [16]. Thermal processing, in particular, can severely alter surface color due to chemical and enzymatic degradation of pigments [16]. In turn, some of these degradations may be minimized in salt processing samples if kept under low temperatures, but conversely, in these treatments, flow of pigments from the food matrix along with the water might occur [9]. 

The color coordinates (CIE L*a*b*) of air-dried and salt-processed *U. rigida* over six months of storage is summarized in Table 2. Note that, in this system, the results are expressed as negative or positive values in relation to a particular color coordinate: a* represents greenish and reddish colors in case of negative and positive values, respectively, while negative and positive b* values are bluish and yellowish tonalities, respectively. The L* coordinate measures the luminosity as an approximation to a greyscale, ranging between black (0) and white (100) [17] and the browning index (BI, estimated by considering a*, b*, and L* parameters) is defined as brown color purity [18].

The greenish tonality of *U. rigida*, just after the application of the treatments and rehydration, was not significantly different among the samples (as reflected by a* values close to −15), regardless, it tended to be less intense in the air-dried ones. In turn, its fade during storage was more pronounced in salt-processed samples. While the mean a* value in dried *Ulva* was slightly lowered in the first 30 days, from −14.78 ± 0.35 to −13.08 ± 0.95, and kept constant for up to six months of storage, those of salt-processed algae continued to decrease until 120 days (brine and salted 28%) or 180 days (salted 40%), reaching values of −10 to −11. The superior impact observed in salt-treated samples is probably partly due to some flow of chlorophylls along with the water and to their degradation, which results in the gray-brown compounds pheophytin or pheophorbide. Degradation of chlorophylls in salt-processed *Ulva* might be favored by their high water content (57%–71%) in comparison to that of air-dried samples (14%–15%), regardless if they were kept under lower temperatures (4 °C). 

As for a*, the b* coordinate in air-dried *U. rigida* was slightly distinct from those in salted-processed samples (values of 42 vs. 39, respectively), suggesting that air-drying could intensify the yellow tonalities of this macroalgae. As well, changes in the b* coordinate during storage were evident in all samples, with ∆ (i.e., variation between t0 and t180) of 5.7, 6.5, and 8–9 in air-dried, salted at 28%, brined and salted at 40%, respectively, overall indicating a clear intensification of yellow tonalities of *U. rigida* in this period. As for other vegetables, this color change is expected to be associated with some changes/degradation of carotenoids, as also a reflection of those occurring in chlorophylls [16]. In fact, the green color of *U. rigida* is mainly due to the presence of high quantities of chlorophylls that mitigate the yellow color of carotenoids, whereas chlorophyll degradation intensifies their yellow coloration [19].

In general, browning coloration results from both enzymatic and non-enzymatic oxidation of phenolic compounds or non-enzymatic Maillard reactions between reducing sugars and amino acids, the latter being particularly promoted at high temperature [16]. Once cell walls and cellular membranes lose their integrity (a fact that might occur because of the water loss during the treatments), enzymatic oxidation proceeds much more rapidly. As can be concluded from Table 2, our results indicated that at t0, the brown tone of air-dried samples was more intense in those treated with salt, a fact that probably results from non- and enzymatic reactions occurring during the drying processing, as previously mentioned. Notably, after air-drying, the browning tone of *Ulva* was maintained constant for at least 30 days. In fact, ΔBI in air-dried samples occurred between t30 and t60 and later from t120 to t180. Conversely, significant changes in BI of salt-processed samples (kept at 4 °C) were visible in the two first months and these seemed to be delayed at a lower water content (salted at 40% in comparison to brine/salted at 28%). This also supports the hypothesis that BI changes during algae storage are dictated by enzymatic browning phenomena.

**Figure 1:**
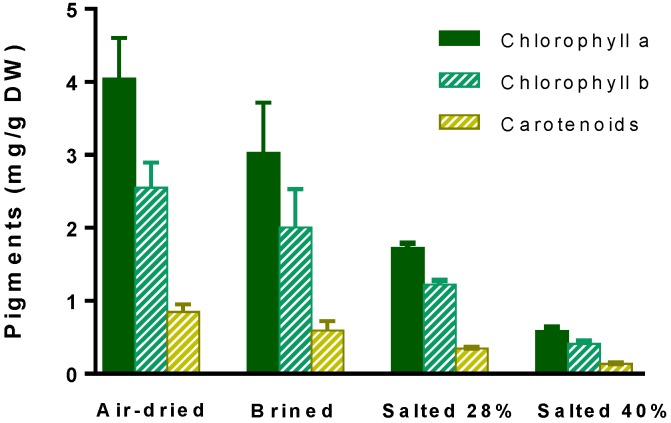
Levels of chlorophyll a, b and total carotenoids extracted from *Ulva rigida* submitted to different preservative processes (air-drying, brining, salting at 28% and 40%) after six months of storage. The macroalgae were stored at room temperature (air-dried) or at 4 °C (salt-processed). The results correspond to mean ± standard deviation (n = 3).

Overall, it is clear that just after the air-drying process at 25 °C, if rehydrated, *U. rigida* is visually more yellow/brown than those treated with salt. Moreover, as expected, the total color difference parameter (calculated on the basis of a*, b*, and L* coordinates) confirmed that changes in colors during the storage time were particularly evident in salt-treated samples, probably due to superior changes in the cells’ structures, which might contribute to additional losses/changes in pigments. In fact, this is partially supported by the results of Figure 1, which show higher levels of extracted chlorophylls and total carotenoids (presumably lutein as shown in our previous work [15]) in air-dried *U. rigida* at t180, when compared to salt-treated samples.

### 2.3. Mechanical Properties

**Figure 2:**
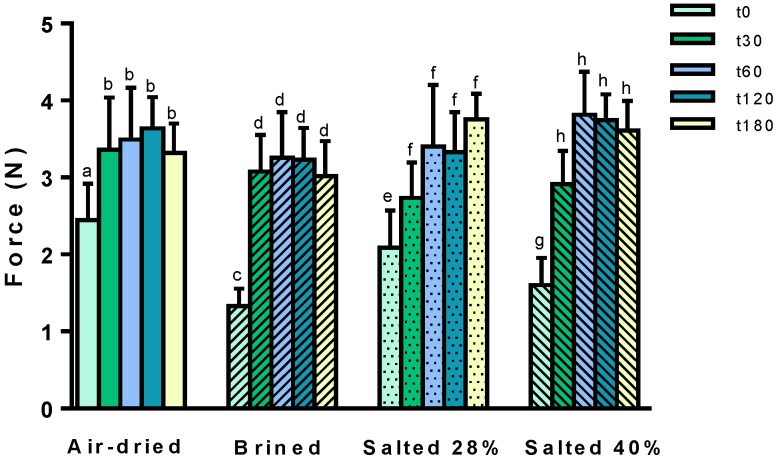
Force (N) required to fracture *Ulva rigida* submitted to different preservative processes (air-drying, brining, salting at 28% and 40%), over six months of storage; t0, t30, t60, t120, t180 correspond to day 0 (i.e., just after the application of the processing treatment), and after storage for 30, 60, 120, and 180 days, respectively. Storage was done at room temperature (air-dried) or at 4 °C (salt-processed). The results correspond to mean ± standard deviation (n > 5). Different letters in a treatment condition indicate significant differences (*p* < 0.05) according to Tukey´s test.

Texture is another physical parameter that is frequently affected by industrial processing, and in the case of *U. rigida*, a change in blade fracturability may occur [20,21]. As depicted in Figure 2, the force required to fracture *U. rigida* blades varied among the samples, but changes were also relevant during storage. At t0, air-dried *U. rigida* presented the highest resistance to fracturing, in comparison to brined and salted at 40% (force values of 2.5 N vs. 1.3–1.6 N) or even those salted at 28% (although differences with the latter were less evident). Overall, the results suggest that for short storage periods, salt-processed *U. rigida* had less fracturability as compared to air-dried samples. Besides, these are also in line with those reported by Prinzivalli et al. [22], who showed that osmotic dehydration or salting decreases the amount of force necessary to perforate vegetable samples.

Over the 180 days of storage, all *U. rigida* showed a tendency to increase their resistance to fracture, which on average reached values close to 3–4 N. Curiously, this increment occurred in a shorter period for air-dried and brined macroalgae when compared to salted samples (1 vs. 2 months). Also note that it is possible that the increment of fracturability of *U. rigida* is partially related to their conservation at low temperatures, since cold is known to contribute to firmness of foods [23].

### 2.4. Nutritional Parameters

*Ulva* sp. are recognized for their richness in fibers (mostly ulvans) and minerals, among which Fe is of most importance, as accumulation is assumed to be much superior than in Rhodophyta and Phaeophyta, reaching values of 6 g/kg dw [24,25]. Alike other green species, they are also a good source of proteins (10%–25% dw), containing considerable levels of essential amino acids [26]. Moreover, despite lipids may in general only represent up to about 5% of the whole algal dry weight, they display an important nutritional value, with emphasis in n-3 polyunsaturated fatty acids (PUFAs) like α-linolenic acid, eicosapentaenoic acid, and docosahexaenoic acid [27,28]. However, as for other natural products, the overall nutritional properties of *Ulva* sp. are dependent on factors which, among others, include specific species, seasonality, conditions, processing, and storage conditions [29,30]. 

In order to evaluate possible differences on the nutritional value of *U. rigida* submitted to distinct processing over storage time, levels of protein, fiber, iron, and of fatty acids (FA) were evaluated just after application of the treatment (day 0, t0) and at the end of six months of storage (t180). In this context, please note that regardless of the nitrogen-to-protein conversion factor of 6.25 being the most commonly used, it overestimates the protein content in seaweeds [31] and because of that, a conversion factor of 5 is more accurate [32]. 

**Figure 3:**
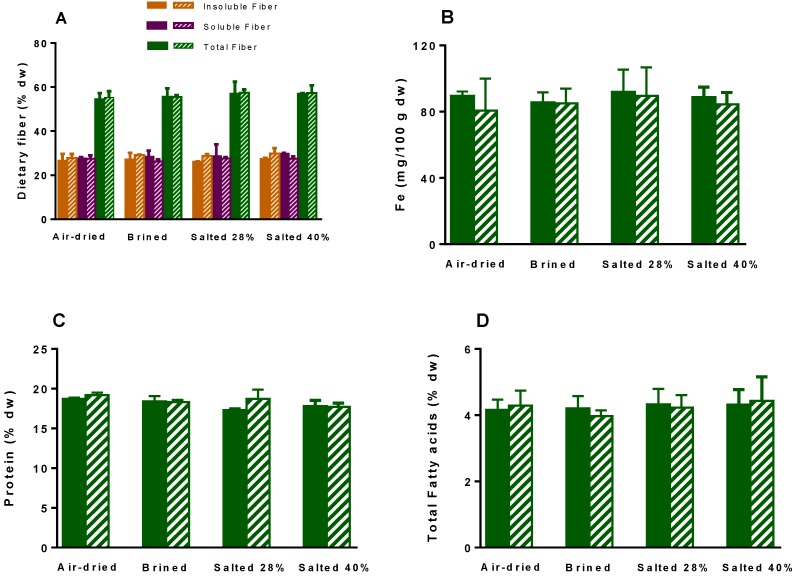
Content of fiber (**A**), iron (**B**), protein, as determined by N x correction factor of 5; (**C**) and total fatty acids (**D**) of *Ulva rigida* submitted to different preservative processes (air-drying, brining, salting at 28% and 40%), just after the application of treatment (t0, full representations) and after six months of storage (t180, line representations) at room temperature (air-dried) or 4 °C (salt-processed). The results correspond to mean ± standard deviation (n = 3).

At t0, all processed samples showed a high content of insoluble and soluble fibers, overall accounting for 55%–57% dw and of proteins (17.5%–19.2% dw), as shown in Figure 3. As well, the amounts of Fe and total FA were close among the distinct samples (86–92 mg/100 g dw and 3.9%–4.3% dw, respectively). Moreover, the gathered results regarding these parameters, at t180, remained close to those at day 0, thus suggesting that nutritional value of air-dried and salt-processed *U. rigida* are not significantly changed over six months (if kept under the herein applied conditions), yet one must note that levels might not reflect specific changes in the nutrients. In this regard, among fibers, proteins, and fatty acids, the latter are the most prone to oxidation processes and were further analyzed as indicators of possible changes due to processing and storage.

Despite the occurrence of slight variations in specific fatty acids amongst the processed samples, they showed an FA composition mainly rich in palmitic (C16:0), palmitoleic (C16:1 *n*-7), oleic (C18:1 *n*-9), linoleic (C18:2), linolenic (C18:3), stearidonic (18:4), and behenic (C22:0) acids, with trace amounts of the omega-3 fatty acids eicosapentaenoic (C20:5) and eicosatetraenoic (C20:4), and an overall low omega-6/omega-3 ratio (0.38–0.56) and unsaturated fatty acid (UFA)/saturated fatty acid (SFA) ratio (0.79–0.94), as shown in Table 3. In general, this profile is in line with reported data for *U. rigida*, despite some differences on the total amount of lipids and/or fatty acids, as well as on the relative abundance of specific FA, a fact that is attributed to the impact of multiple factors (growth conditions, seasonal effects, processing, and others) on the chemical composition of algae, as well as to the distinct analytical methods applied [31,32,33]. Notably, in general, the relative abundance of *U. rigida* FA at t180 was not significantly different from that at t0, thus indicating that FA were kept stable during the storage period of six months both in dried and salt-processed samples. 

## 3. Materials and Methods 

### 3.1. Sample Collection and Treatments 

*U. rigida* was produced by ALGAplus Lda. (production site located at Ria de Aveiro coastal lagoon, Northern Portugal, 40°36′43″ N, 8°40′43″ W), in an open land-based integrated multi-trophic aquaculture (IMTA) system. After hand collection in November 2016, the macroalgae batch was treated according to internal procedures of ALGAplus. Samples were washed with filtered and sterilized (UV and ozone) seawater from Ria de Aveiro, followed by centrifugation to remove excess water. A portion of this batch was dried at 25 °C for 16 h in an industrial convective dryer (customized built by ALGAplus Lda.) and then stored in multilayer paper-plastic bags and kept at the ALGAplus facility in a non-climatized room until analysis, while the remaining parts of the batch were processed by salt-treatments. For brining, 4 kg of *U. rigida* was submerged in a 25% (*w*/*v*) solution of kitchen salt for 5 min, while dry-salting consisted of mixing *U. rigida* with kitchen salt at 28% (*w*/*w*) or at 40% (*w*/*w*). All the salt-processed samples were also stored at ALGAplus, in covered Styrofoam boxes at 4 °C until analysis. 

The effect of air-drying or salt-processing on moisture content, superficial color, and mechanical properties of *U. rigida* were assessed at five distinct points during the storage period, namely at day 0, 30, 60, 120, and 180 (t0, t30, t60, t120, and t180, respectively). The nutritional parameters were compared at t0 and t180. After reception at the laboratory, salt was manually removed (if salt-processed). Samples were rehydrated in distilled water for 15 min and then evaluated for color and fracturability. For the nutritional parameters and pigments extraction, rehydrated samples were frozen, freeze-dried, ground (Yellowline A10 mill, 20,000 rpm, IKA, Works Inc., Wilmington, NC, USA) and sieved with <0.25 mm pore sieve.

### 3.2. Surface Color 

Rehydrated seaweeds were cleaned of excess water with absorbent paper and surface color was measured with a colorimeter (CM 2300d, Konica Minolta, Japan) through coordinates CIELAB a* (+ red, − green), b* (+ yellow, − blue), and L* (lightness) [16] and the color difference (∆E*) was calculated by the equation:

∆E* = [(a* − a0*)^2^ + (b* − b0*)^2^ + (L* − L0*)^2^]1/2 (1)

where a*, b*, and L* correspond to the coordinates of processed macroalgae, while a0*, b0*, and L0* correspond to T0. These coordinates were used in browning index (BI) determination [34], through:

BI = [100 × (X − 0.31)]/0.17 where X = (a* + 1.75 × L*)/(5.645 × L* + a* − 3.012 × b*).(2)

### 3.3. Texture

The fracturing of the samples was evaluated using a texturometer TA-HDi (Stable Micro Systems), with a 5 kg cell and a 6 mm piercing probe of stainless steel. *Ulva rigida* was soaked in water for 15 min and placed on the analysis platform. The peak of the strength needed to pierce the samples was registered by the software Texture Expert Exceed 2.64 and expressed in Newtons (N).

### 3.4. Chemical Composition

#### 3.4.1. Contents of chlorophylls and carotenoids

Chlorophylls and carotenoids were extracted with acetone with 1% of butylated hydroxytoluene (BHT) for 24 h, using powdered samples and a mass/volume ratio of 1:100. The extraction solution was filtered through a nylon filter of 0.45 µm (Whatman™, Buckinghamshire, UK). Absorbance was measured against a blank of acetone with 1% BHT with a wavelength range of 400 to 700 nm. The total amount of chlorophyll-a, chlorophyll-b, and carotenoids were then calculated according to the formulas of Lichtenthaler [35].

#### 3.4.2. Moisture Content

Two grams of *U. rigida* were placed in previously dried crucibles (2 h, 105 °C). The samples were dried in an oven at 105 °C for 10–12 h and weight was registered after cooling (30 min).

#### 3.4.3. Protein Content

The nitrogen content of samples was determined by elemental analysis using a LECO TruSpec-Micro CHNS 630-200-200 elemental analyzer (St. Joseph, MI, USA) at a combustion furnace temperature of 1075 °C and an afterburner temperature of 850 °C. Nitrogen was detected by thermal conductivity. Protein content was calculated using a nitrogen–protein conversion factor of 6.25. 

#### 3.4.4. Dietary Fiber

Macroalgae were analyzed in terms of their insoluble, soluble, and total dietary fiber content, according to the enzymatic gravimetric method AOAC 991.43. This analysis was performed using the Total Dietary Fiber Assay kit (Megazyme, Bray, UK).

#### 3.4.5. Iron (Fe)

A microwave assisted acid digestion procedure was performed for sample mineralization according to Domínguez-González et al. [36], with some modifications. Briefly, dried samples (ca. 200–220 mg) were accurately weighted into acid-washed Teflon vessels and were added with 2 mL HNO_3_ 69% (*w*/*w*). Then, the vessels were closed and placed inside a microwave oven to be digested over 2 cycles of the following extraction program: temperature was first raised to 170 °C (ramp time: 5 min) and held for 10 min. After cooling down, the vessels were carefully opened and 0.25 mL H_2_O_2_ 30% (*w*/*w*) were added, followed by a second microwave digestion cycle. Fe was quantified in a Perkin Elmer (Waltham, MA, USA) Analyst 100 flame atomic absorption spectrometer equipped with single hollow cathode lamps for each element and an air-acetylene burner. 

#### 3.4.6. Fatty Acid Profile

The fatty acid profile was analyzed by GC-MS after their conversion to fatty acid methyl esters (FAME) following the methodology of O’Fallon et al. [37]. Samples were analyzed on a gas chromatograph mass spectrometer GCMS-QP2010 (Shimadzu, Kyoto, Japan) equipped with an AOC-20i auto-injector and a DB-5 ms column (30 m × 0.25 mm diameter, 0.25 µm thickness). The equipment operated under the following conditions: initial temperature, 70 °C for 5 min; temperature gradient, 4 °C min^−1^; final temperature, 250 °C; temperature gradient, 2 °C min^−1^; final temperature, 300 °C for 5 min; injection temperature, 320 °C; split ratio, 100:0. Identification of FAME was obtained by co-chromatography with authentic commercially available FAME standards (Supelco™ 37 Component FAME Mix, catalogue no. 47885-U, Supelco, Bellefonte, PA, USA). Total FAME content was quantified by comparison with a known amount of added nonadecanoic acid 19:0 (Thermo Fisher Scientific, Kandel, Germany) as internal standard. The internal standard (1000 mL, 2 mg mL^−1^) was added prior to direct transmethylation to the ground seaweed powder.

### 3.5. Statistics

Results were reported on a dried matter basis and expressed as mean ± standard deviation, except for the fatty acid profile where only the mean was presented. At least three replicates were assessed for all the determinations. Data were statistically analyzed by a trial version of GraphPad Prism 6.01 software (OriginLab Corporation, Northampton, MA, USA) using two-way ANOVA and Tukey-HSD multiple comparisons test (*p* < 0.05). 

## 4. Conclusions

The effect of different processing and storage treatments of *U. rigida* were investigated for several aspects, namely moisture, surface color, texture, and nutritional parameters. During the 180 days of storage at room temperature (air-dried) or at 4 °C (dry-salted and brined), the algae increased their resistance to fracture and, particularly those submitted to salting, intensified their yellowish/brownish tonalities, a fact that was reflected by the lower amounts of recovered chlorophylls and carotenoids. Yet in general, the amount of insoluble and soluble fibers, protein, and Fe were highly representative and remained stable during storage. A similar trend was also observed for the fatty acids profile, which was mainly rich in palmitic acid, but also in unsaturated fatty acids (44%–49% total fatty acids) and overall characterized by a low omega 6/omega 3 ratio. The gathered data suggests that air-drying at 25 °C, brining at 25%, and dry-salting at 28% and 40% enables the nutritional value of *U. rigida* to be kept over long periods, if kept under suitable conditions, albeit losses of some phytochemicals such as chlorophylls and carotenoids might occur, particularly in dry-salted samples. 

## Figures and Tables

**Table 1:** Moisture content of *Ulva rigida* submitted to different preservative processes (air-drying, brining, salting at 28% and 40%), over storage up to 180 days.

Conditions	Moisture Content (%)				
	t0	t30	t60	t120	t180
**Air-dried**	14.22 ± 0.37 ^a^	14.76 ± 0.31 ^a^	15.50 ± 0.09 ^a^	15.01 ± 0.09 ^a^	14.47 ± 0.13 ^a^
**Brined**	71.37 ± 0.36 ^b^	70.09 ± 2.05 ^b^	68.17 ± 0.97 ^b^	69.40 ± 1.31 ^b^	70.12 ± 1.52 ^b^
**Salted 28%**	70.86 ± 5.05 ^c^	61.92 ± 1.03 ^d^	61.43 ± 2.49 ^d^	62.63 ± 1.58 ^d^	62.98 ± 1.01 ^d^
**Salted 40%**	60.08 ± 1.57 ^e^	62.54 ± 3.79 ^e^	57.32 ± 2.50 ^e^	58.62 ± 1.56 ^e^	58.05 ± 2.44 ^e^

t0, t30, t60, t120, t180 correspond to day 0 (i.e., just after the application of the processing treatment), and after storage for 30, 60, 120, and 180 days, respectively. Storage was done at room temperature (air-dried) or 4 °C (salt-processed). Values are presented as mean ± standard deviation, n = 3. Different letters in the line indicate significant differences (*p* < 0.05) according to Tukey’s test.

**Table 2:** Surface color parameters of *Ulva rigida* submitted to different preservative processes (air-drying, brining, salting at 28% and 40%), over six months of storage.

CIELAB	Treatment	t0	t30	t60	t120	t180
a*	Air-dried	−14.78 ± 0.35 ^a^	−13.08 ± 0.95 ^a^	−13.35 ± 0.79 ^a^	−13.28 ± 1.13 ^a^	−13.16 ± 0.81 ^a^
	Brined	−15.42 ± 1.10 ^b^	−14.02 ± 1.09 ^b^	−12.63 ± 1.10 ^c^	−10.75 ± 0.96 ^c^	−10.35 ± 1.23 ^c^
	Salted 28%	−15.19 ± 1.06 ^d^	−14.59 ± 1.46 ^d^	−14.26 ± 1.72 ^d^	−11.25 ± 1.03 ^e^	−11.20 ± 1.95 ^e^
	Salted 40%	−15.14 ± 0.26 ^f^	−14.94 ± 0.77 ^f^	−13.35 ± 0.51 ^f^	−12.42 ± 1.41 ^f^	−10.69 ± 1.37 ^f^
b*	Air-dried	41.71 ± 2.58 ^a^	40.84 ± 1.09 ^a^	43.94 ± 3.13 ^a^	44.34 ± 2.97 ^a^	47.41 ± 2.86 ^b^
	Brined	38.58 ± 2.04 ^c^	43.80 ± 2.06 ^d^	43.94 ± 1.35 ^d^	43.12 ± 0.87 ^d^	46.94 ± 1.99 ^d^
	Salted 28%	39.14 ± 3.07 ^e^	41.59 ± 2.52 ^e^	44.75 ± 2.46 ^f^	43.28 ± 2.66 ^f^	45.64 ± 2.48 ^f^
	Salted 40%	38.13 ± 2.46 ^g^	42.52 ± 2.56 ^h^	43.60 ± 4.19 ^h^	43.95 ± 1.70 ^h^	46.91 ± 1.72 ^h^
L*	Air-dried	51.33 ± 1.58 ^a^	49.35 ± 3.38 ^a^	50.34 ± 3.10 ^a^	50.40 ± 2.78 ^a^	52.63 ± 2.51 ^a^
	Brined	48.78 ± 1.12 ^b^	50.68 ± 1.75 ^b^	51.02 ± 1.59 ^b^	49.86 ± 1.40 ^b^	54.50 ± 2.04 ^c^
	Salted 28%	48.85 ± 1.34 ^d^	48.10 ± 1.67 ^d^	50.76 ± 2.24 ^d^	50.77 ± 2.92 ^d^	53.46 ± 2.65 ^e^
	Salted 40%	48.65 ± 2.48 ^f^	49.75 ± 2.12 ^f^	50.78 ± 3.41 ^f^	51.04 ± 2.00 ^f^	54.61 ± 2.22 ^g^
**BI**	Air-dried	115.51 ± 6.37 ^a^	113.17 ± 7.62 ^a^	137.93 ± 9.24 ^b^	139.36 ± 11.08 ^b^	146.23 ± 10.96 ^b^
	Brined	105.18 ± 6.14 ^c^	130.46 ± 7.98 ^d^	138.30 ± 5.31 ^d^	135.27 ± 6.66 ^d^	138.34 ± 11.69 ^d^
	Salted 28%	105.04 ± 11.81 ^e^	127.25 ± 12.54 ^f^	136.00 ± 6.70 ^f^	131.87 ± 7.58 ^f^	133.74 ± 10.39 ^f^
	Salted 40%	108.19 ± 12.95 ^g^	123.27 ± 5.72 ^g^	136.61 ± 13.74 ^h^	133.58 ± 8.03 ^h^	136.49 ± 5.64 ^h^
ΔE*	Air-dried	^_^	3.91 ^a^	4.46 ^a^	4.90 ^a^	5.81 ^a^
	Brined	^_^	5.79 ^b^	6.38 ^b^	6.40 ^b^	11.45 ^c^
	Salted 28%	^_^	3.77 ^d^	6.55 ^d^	6.91 ^d^	9.21 ^e^
	Salted 40%	^_^	6.16 ^f^	7.74 ^f^	7.86 ^f^	12.12 ^g^

BI—browning index; ΔE—total color difference; t0, t30, t60, t120, t180 correspond to day 0 (i.e., just after the application of the processing treatment), and after storage for 30, 60, 120, and 180 days, respectively. Storage was done at room temperature (air-dried) or 4 °C (salt-processed). Values are presented as mean ± standard deviation, n = 3 (except for ΔE, which corresponds to the mean value). Different letters in the line indicate significant differences (*p* < 0.05) according to Tukey´s test.

**Table 3:** Fatty acid composition (relative abundance (%) of total) of *Ulva rigida* submitted to different preservative processes (air-drying, brining, salting at 28% and 40%) just after the application of treatment (t0) and after six months of storage (t180).

	Air-Dried		Brined		Salted 28%		Salted 40%	
	t0	t180	t0	t180	t0	t180	t0	t180
*Saturated*								
C14:0	6.44 ± 0.26 ^a^	5.37 ± 0.20 ^b^	7.33 ± 0.02 ^a^	7.33 ± 0.25 ^a^	6.37 ± 0.03 ^a^	7.21 ± 0.10 ^b^	6.52 ± 0.14 ^a^	6.07 ± 0.03 ^a^
C16:0	29.87 ± 0.11 ^a^	30.44 ± 0.13 ^a^	32.32 ± 0.31 ^a^	31.87 ± 0.80 ^a^	29.99 ± 0.20 ^a^	31.20 ± 0.31 ^a^	30.15 ± 0.17 ^a^	31.19 ± 0.64 ^a^
C18:0	4.40 ± 0.35 ^a^	4.99 ± 0.64 ^a^	5.27 ± 0.00 ^a^	4.82 ± 0.56 ^a^	5.01 ± 0.25 ^a^	5.54 ± 0.91 ^a^	4.70 ± 0.85 ^a^	4.81 ± 0.77 ^a^
C22:0	10.86 ± 0.30 ^a^	11.11 ± 0.03 ^a^	11.07 ± 0.32 ^a^	11.87 ± 0.06 ^a^	10.71 ± 0.02 ^a^	10.74 ± 0.22 ^a^	10.56 ± 0.33 ^a^	10.02 ± 0.36 ^a^
*Unsaturated*								
C16:1 (*n*-7)	10.14 ± 0.45 ^a^	9.88 ± 0.25 ^a^	10.52 ± 0.08 ^a^	10.67 ± 0.16 ^a^	9.92 ± 0.04 ^a^	10.25 ± 0.49 ^a^	9.80 ± 0.40 ^a^	9.77 ± 0.53 ^a^
C18:1 (*n*-9)	10.39 ± 0.23 ^a^	11.07 ± 0.19 ^a^	11.13 ± 0.24 ^a^	9.81 ± 0.13 ^b^	11.26 ± 0.09 ^a^	10.85 ± 0.06 ^a^	10.48 ± 0.23 ^a^	11.65 ± 0.29 ^b^
C18:2 (*n*-6)	8.80 ± 0.32 ^a^	8.32 ± 0.34 ^a^	7.98 ± 0.43 ^a^	8.10 ± 0.25 ^a^	7.80 ± 0.02 ^a^	7.83 ± 0.91 ^a^	7.70 ± 0.57 ^a^	7.27 ± 0.41 ^a^
C18:3 (*n*-3)	9.21 ± 1.05 ^a^	9.59 ± 0.21 ^a^	7.23 ± 0.96 ^a^	7.87 ± 0.72 ^a^	9.73 ± 0.24 ^a^	8.31 ± 2.53 ^a^	10.75 ± 1.02^a^	10.37 ± 1.10 ^a^
C18:4 (*n*-3)	9.88 ± 0.59 ^a^	9.23 ± 0.20 ^a^	7.15 ± 0.23 ^a^	7.67 ± 0.35 ^a^	9.21 ± 0.02 ^a^	8.05 ± 0.09 ^a^	9.32 ± 0.31 ^a^	8.84 ± 0.27 ^a^
C20:4 (*n*-6)	d	d	d	d	d	d	d	d
C20:5 (*n*-3)	d	d	d	d	d	d	d	d
∑ SFA	50.58 ± 1.01 ^a^	51.91 ± 1.00 ^a^	55.99 ± 0.65 ^a^	55.89 ± 1.67 ^a^	52.07 ± 0.50 ^a^	54.70 ± 1.53 ^a^	51.94 ± 1.49 ^a^	52.09 ± 1.80 ^a^
∑ MUFA	20.53 ± 0.67 ^a^	20.95 ± 0.44 ^a^	21.65 ± 0.31 ^a^	20.47 ± 0.20 ^a^	21.18 ± 0.13 ^a^	21.10 ± 0.55 ^a^	20.29 ± 0.63 ^a^	21.42 ± 0.82 ^a^
∑ PUFA	28.89 ± 1.96 ^a^	27.14 ± 0.74 ^a^	22.36 ± 1.62 ^a^	23.64 ± 1.32 ^a^	26.74 ± 0.27 ^a^	24.20 ± 3.52 ^a^	27.78 ± 1.91 ^a^	26.48 ± 1.79 ^a^
UFA/SFA	0.94	0.93	0.79	0.79	0.92	0.83	0.93	0.92
Ω6/Ω3	0.44	0.44	0.56	0.52	0.41	0.48	0.38	0.38

Different letters in a treatment condition indicate significant differences (*p* < 0.05) according to Tukey´s test. In order, C14:0 (myristic acid); C16:0 (palmitic acid); C18:0 (stearic acid); C22:0 (behenic acid); C16:1 (palmitoleic acid); C18:1 (oleic acid); C18:2 (linoleic acid); C18:3 (linolenic acid); C18:4 (stearidonic acid); C20:4 (eicosatetraenoic acid); C20:5 (eicosapentaenoic acid). SFA—saturated fatty acids; MUFA—monounsaturated fatty acids; PUFA—polyunsaturated fatty acids; UFA—unsaturated fatty acids; d—detected.
